# The Examiner for the Coming Year

**Published:** 1874-12-01

**Authors:** 


### THE EXAMINER FOR THE COMING YEAR.

THE present number of the EXAMINER is presented to our readers enlarged and in a new outside dress.

For three years past our volumes have contained more reading matter than any other three dollar journal in the country. A constant and steady increase in our subscription list has enabled us with the commencement of each year to increase somewhat the size, as well as to improve the style and character of our issues. For the coming year the semi-monthly numbers will be similar to the present, containing each thirty pages of reading matter. Sixty of our double column pages per month is fully equal to eighty pages of the ordinary octavo form. For any of the other medical periodicals giving this amount of reading matter the subscription price is placed at four or five dollars.

We have an active and efficient corps of assistants and contributors in the various departments, and shall commence the coming year better prepared than ever before to maintain the value and interest of our publication.

In order to sustain the very considerable increase in expenditure we ask, and confidently expect, that *all* of our *old friends and subscribers* will promptly renew their subscriptions at or before the commencement of the new year.

We shall adhere strictly to the system of advance payment of subscriptions in all cases. A failure to renew, or to order the continuance of subscriptions after their expiration, is considered equivalent to a discontinuance. This is the only system upon which a periodical can be efficiently or successfully conducted, and in the end is much more satisfactory to the subscribers as well as the publishers.

Many new subscriptions for the coming volume are already arriving and we hope to commence the new year with an increase of at least one-half in our circulation. Let all of our friends speak a good word for the EXAMINER to the physicians in their own neighborhood, and they will easily obtain for us two or three new subscribers.

In our announcement of club rates we offer any of the three dollar journals at two-fifty; the four dollar journals at three; and the five dollar publications at four dollars, in connection with the EXAMINER. Those physicians who have been in the habit of subscribing for two, three or more journals, at full rates, will thus be enabled to save sufficient from the other subscriptions to make up the price of our journal.

We would also call attention to the fact that the new postal law, which goes into effect January 1st, requires us to prepay postage on all numbers sent to subscribers. The postage for the year will amount to fifteen cents. This amount we shall charge on to the subscription, and subscribers will please bear in mind the fact in forwarding their money.

